# Actionable pharmacogenetic variants in Hong Kong Chinese exome sequencing data and projected prescription impact in the Hong Kong population

**DOI:** 10.1371/journal.pgen.1009323

**Published:** 2021-02-18

**Authors:** Mullin Ho Chung Yu, Marcus Chun Yin Chan, Claudia Ching Yan Chung, Andrew Wang Tat Li, Chara Yin Wa Yip, Christopher Chun Yu Mak, Jeffrey Fong Ting Chau, Mianne Lee, Jasmine Lee Fong Fung, Mandy Ho Yin Tsang, Joshua Chun Ki Chan, Wilfred Hing Sang Wong, Jing Yang, William Chun Ming Chui, Patrick Ho Yu Chung, Wanling Yang, So Lun Lee, Godfrey Chi Fung Chan, Paul Kwong Hang Tam, Yu Lung Lau, Clara Sze Man Tang, Kit San Yeung, Brian Hon Yin Chung

**Affiliations:** 1 Department of Paediatrics and Adolescent Medicine, LKS Faculty of Medicine, The University of Hong Kong, Pokfulam, Hong Kong SAR, China; 2 Department of Pharmacy, Queen Mary Hospital, Pokfulam, Hong Kong SAR, China; 3 Department of Surgery, LKS Faculty of Medicine, The University of Hong Kong, Pokfulam, Hong Kong SAR, China; 4 Department of Paediatrics and Adolescent Medicine, Duchess of Kent Children's Hospital, Pokfulam, Hong Kong SAR, China; 5 Department of Paediatrics and Adolescent Medicine, Queen Mary Hospital, Pokfulam, Hong Kong SAR, China; 6 Department of Paediatrics and Adolescent Medicine, The Hong Kong Children’s Hospital, Kowloon Bay, Hong Kong SAR, China; 7 Dr Li Dak-Sum Research Centre, The University of Hong Kong–Karolinska Institutet Collaboration in Regenerative Medicine, Pokfulam, Hong Kong SAR, China; University of Alabama at Birmingham, UNITED STATES

## Abstract

Preemptive pharmacogenetic testing has the potential to improve drug dosing by providing point-of-care patient genotype information. Nonetheless, its implementation in the Chinese population is limited by the lack of population-wide data. In this study, secondary analysis of exome sequencing data was conducted to study pharmacogenomics in 1116 Hong Kong Chinese. We aimed to identify the spectrum of actionable pharmacogenetic variants and rare, predicted deleterious variants that are potentially actionable in Hong Kong Chinese, and to estimate the proportion of dispensed drugs that may potentially benefit from genotype-guided prescription. The projected preemptive pharmacogenetic testing prescription impact was evaluated based on the patient prescription data of the public healthcare system in 2019, serving 7.5 million people. Twenty-nine actionable pharmacogenetic variants/ alleles were identified in our cohort. Nearly all (99.6%) subjects carried at least one actionable pharmacogenetic variant, whereas 93.5% of subjects harbored at least one rare deleterious pharmacogenetic variant. Based on the prescription data in 2019, 13.4% of the Hong Kong population was prescribed with drugs with pharmacogenetic clinical practice guideline recommendations. The total expenditure on actionable drugs was 33,520,000 USD, and it was estimated that 8,219,000 USD (24.5%) worth of drugs were prescribed to patients with an implicated actionable phenotype. Secondary use of exome sequencing data for pharmacogenetic analysis is feasible, and preemptive pharmacogenetic testing has the potential to support prescription decisions in the Hong Kong Chinese population.

## Author summary

Pharmacogenetic testing provides relevant drug phenotype information to guide personalized drug prescription, which potentially improves drug efficacy and prevent adverse drug reactions. However, its implementation in the Chinese population is limited by the lack of Chinese-specific pharmacogenetics data. In this study, we studied the spectrum of 133 actionable pharmacogenetic variants and rare deleterious variants in 108 pharmacogenes using an exome sequencing consisting of 1116 Hong Kong Chinese subjects. It was found that nearly all individuals carried at least one actionable pharmacogenetic variant and one rare, predicted deleterious pharmacogenetic variant. In addition, we projected the potential prescription impact of actionable pharmacogenetic variants using prescription data of the Hong Kong's public healthcare system. We estimated that one-seventh of the Hong Kong population received at least one of the 36 drugs with clinical pharmacogenetics guideline recommendations. The findings demonstrated the potential of pharmacogenetic testing in improving patient care and resource allocation in Chinese. The cohort dataset also supports clinical implementation of pharmacogenetics in the Chinese population.

## Introduction

Pharmacogenetics is the study of variability in drug response caused by genetic variations [1]. It is estimated that over two million hospitalized patients develop severe adverse drug reactions in the United States annually, incurring a direct medical cost of 200 billion US dollars (USD) [2]. This indicates a huge potential in reducing healthcare costs even if only a small proportion of adverse drug reactions are preventable by genotype-guided prescription. The current clinical applications of pharmacogenetics are mostly limited to reactive pharmacogenetic testing, in which investigations are ordered only when certain high-risk medications are prescribed, or after an adverse drug reaction has occurred. In contrast, preemptive pharmacogenetic testing allows the optimization of dosing based on genotype information at the time of prescription, minimizing the risk of undesired outcomes [3].

The pharmacogenetics community has established open-access platforms to facilitate the implementation of clinical pharmacogenetics. The Clinical Pharmacogenetics Implementation Consortium (CPIC) provides peer-reviewed, evidence-based guidelines for individual gene-drug combinations to assist clinicians to incorporate pharmacogenetics into daily practice [4]. The Pharmacogenomics Knowledgebase (PharmGKB) provides a multitude of pharmacogenetic information and assigns a level of evidence on variant-medication pairs based on the quantity and quality of available evidence [5,6]. PharmGKB level 1A/B represents the highest level of evidence, which is either supported by large-scale studies or is widely accepted by the pharmacogenetics community. Multiple large population pharmacogenetics studies revealed that nearly all subjects (96.2–97.0%) had at least one actionable pharmacogenetic variant, with a median of two to three [7,8]. PharmGKB level 2A/B annotations are assigned to variant-drug combinations with moderate evidence of association, whereas levels 3 and 4 annotations are assigned to variants with low and preliminary levels of evidence, respectively [6]^.^ The study of pharmacogenetic variants is often limited by sample size. Many actionable pharmacogenetic variants are known because they are relatively common in the population, or their associated adverse reactions are severe. In contrast, the effects of rare variants are largely unknown, but they should not be neglected since they are consistently found in the population and have been predicted to account for nearly all inter-individual variability in more than half of the known pharmacogenes [7,9–12]. Therefore, the study of rare pharmacogenetic variants is important as it can potentially improve the prediction of drug responses.

It is known that pharmacogenetic variations exist across different ethnicities. Currently, Chinese pharmacogenetic data is limited. For example, Asians account for 2% of the eMERGE-PGRNseq cohort [7]. The first large-scale analysis of actionable pharmacogenetic variants in Chinese was published only recently, yet the study did not examine rare variants and its prescription pattern analysis was performed based on data from a children hospital [13]. Besides, the Southern Chinese sub-population accounted for 22.4% of the study subjects and was underrepresented [13]. To address these issues, we examined the spectrum of 133 actionable pharmacogenetic variants and rare deleterious variants in 108 pharmacogenes using an exome sequencing cohort consisting of 1116 Hong Kong (HK) Chinese subjects, who are representative of the Southern Chinese subpopulation. In addition, the potential prescription impact of actionable pharmacogenetic variants was projected on the HK population.

## Materials and methods

### Ethics statement

Written informed consent was obtained for each participant, and this study was approved by the HKU/HA HK West Institutional Review Board (UW12-211, UW12-383, UW 05–282 T/945, UW 12–382, UW 12–469).

### Subjects and exome sequencing (S1 Fig)

A total of 1,141 unrelated, self-reported Chinese were enrolled for exome sequencing for rare disease diagnosis or complex disease research from 2012 to 2019. Exome sequencing was performed on genomic DNA derived from peripheral blood or buccal mucosa by Illumina sequencing platforms, and different exome capture kits were used (S1 Table). The processing of raw exome sequencing data is described in detail in the Supplementary Methods (S1 Text). Briefly, variant calling was performed using a pipeline based on the Genome Analysis Toolkit (GATK), and human leukocyte antigen (HLA) typing was performed using HLA typing from High-quality Dictionary (HLA-HD) [14,15]. The exome sequencing dataset was subjected to stringent quality control (QC) procedures at the sample, variant, and genotype levels and the output data were annotated using wANNOVAR [16]. To avoid over-representation of disease-associated variants, the samples collected from subjects with respiratory diseases and neuromuscular disorders were removed for *CFTR* and *RYR1* analysis, respectively. In this study, a rare variant was defined as a variant having a Genome Aggregation Database (gnomAD) global allele frequency (AF) <1%. A missense variant was considered deleterious when it possessed a Phred-scaled Combined Annotation Dependent Depletion (CADD) score ≥20 [17], or Rare Exome Variant Ensemble Learner (REVEL) score ≥0.7, or PREDICT score ≥0.6; whereas a loss-of-function (LoF) variant was considered deleterious when it possessed a Phred-scaled CADD score ≥20 or a Loss-Of-Function Transcript Effect Estimator (LOFTEE) of “high-confidence” [18–20].

### Selection of actionable pharmacogenetic variants and high-confidence pharmacogenes

To examine the spectrum of known actionable pharmacogenetic variants, those with clinical annotations of PharmGKB level 1A or 1B were selected since they represent variants with the highest level of evidence for clinical actionability of gene-drug associations [5,6,21]. In addition, the literature was reviewed to identify Chinese-specific pharmacogenetic variants that were actionable based on the CPIC guideline [22,23]. This review resulted in a list of 133 actionable pharmacogenetic variants and HLA alleles in 19 genes (S2 Table). To analyze rare pharmacogenetic variants that are potentially actionable, genes with at least one PharmGKB level 1 or 2 clinical annotation were selected, resulting in a list of 108 genes that were considered “high confidence pharmacogenes” (S3 Table) [5,6].

### Projected prescription impact of actionable pharmacogenetic variants in Hong Kong

HK is a city in Southern China with a population of 7.5 million, of which 92% are ethnic Han Chinese [24]. In HK, over 90% of all secondary and tertiary health services are provided by the public healthcare system under the management of the Hospital Authority (HA) [25,26]. All public healthcare service data, including drug prescriptions in the inpatient and outpatient settings, are available in the Clinical Data Analysis and Reporting System (CDARS) database in an unlisted and anonymous manner.

Based on CPIC gene-drug annotations, 44 drugs were matched with known actionable pharmacogenetic variants observed in the exome dataset. Under the public healthcare system in 2019, 36 of these drugs were dispensed by pharmacies and had available data, and eight drugs were not prescribed. All patients in HK who had a prescription record of one or more of the 36 drugs at one or more of the hospitals under the HA between January 1, 2019 and December 31, 2019 were identified from the CDARS database. The number of subjects, drug quantity, and unit cost of each drug were retrieved for the estimation of prescription impact and drug expenditure. Duplications from multiple prescriptions within the same year, different routes of administration and doses for each drug were removed to identify the unique number of subjects. Pharmacophenotypes were assigned based on the genotype definition stated in the CPIC guideline. Phenotypes with at least one prescription recommendation based on the CPIC guideline were considered as actionable. The frequency of each actionable phenotype was derived from the allele frequency of the Hong Kong Chinese exome dataset based on the Hardy-Weinberg equation, except for *CYP2D6*. The frequency of actionable phenotype of *CYP2D6* was retrieved from a published Hong Kong study, since exome sequencing cannot detect haplotype and copy number variations accurately [27]. The projected prescription impact for each drug was estimated by multiplying the frequency of actionable phenotype by the total number of subjects for each individual drug retrieved from CDARS. Drug expenditures were converted from HK dollars (HKD) to USD based on the conversion rate of 7.8 HKD = 1.0 USD.

## Results

### Exome sequencing data characteristics

**Fig 1:**
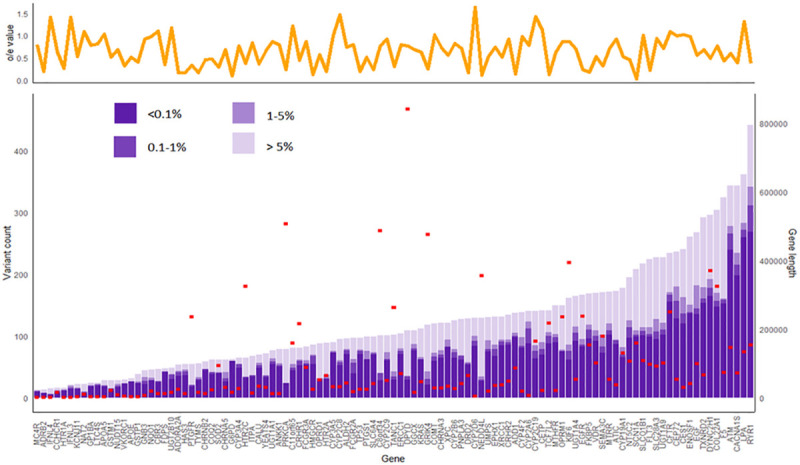
Allele frequency of variants within the 108 pharmacogenes in the dataset. In the upper panel, the yellow line graph shows the gnomAD loss-of-function constraint metric (o/e score) of the respective genes. In the lower panel, the purple bars denote the variant counts in the 108 high-confidence pharmacogenes, while the red rectangles indicate respective gene transcript lengths. Consistent across genes, most variants belong to the very rare category (AF <0.1%). The relationship between variant count, gene transcript length, and constraint (o/e score reported in gnomAD) was analyzed using multiple linear regression analysis. There was significant association between gene transcript length and total variant count (P = 0.0073). In general, the number of variants increased by 0.17 for every kilobase increase in gene length, although outliers existed. In the highly polymorphic gene *CYP2D6*, 29.5 variants were observed for every kilobase of gene length.

A total of 1,116 samples, including 622 males and 494 females, passed the sample-level QC procedures. Among the 108 high confidence pharmacogenes, 104 genes had at least 8X mean coverage in >75% of the samples (S2 Fig). The exceptions were *CCHCR1*, *TNF*, *IFNL4*, and *GSTM1*. A total of 13,165 variants were identified in the 108 pharmacogenes, among which 11,415 were non-coding, 1,719 were exonic, and 31 were canonical splice site variants (S3 Fig). Of all variants identified, 3,501 (26.6%) have never been reported in public databases including gnomAD, dbSNP, and ClinVar (S4 Table). A significant linear relationship between gene transcript length and total number of variants in each gene (p = 0.0073) was observed, with an increase of 0.17 variants per kilobase of gene length (Fig 1).

### Spectrum of known actionable pharmacogenetic variants

**Fig 2:**
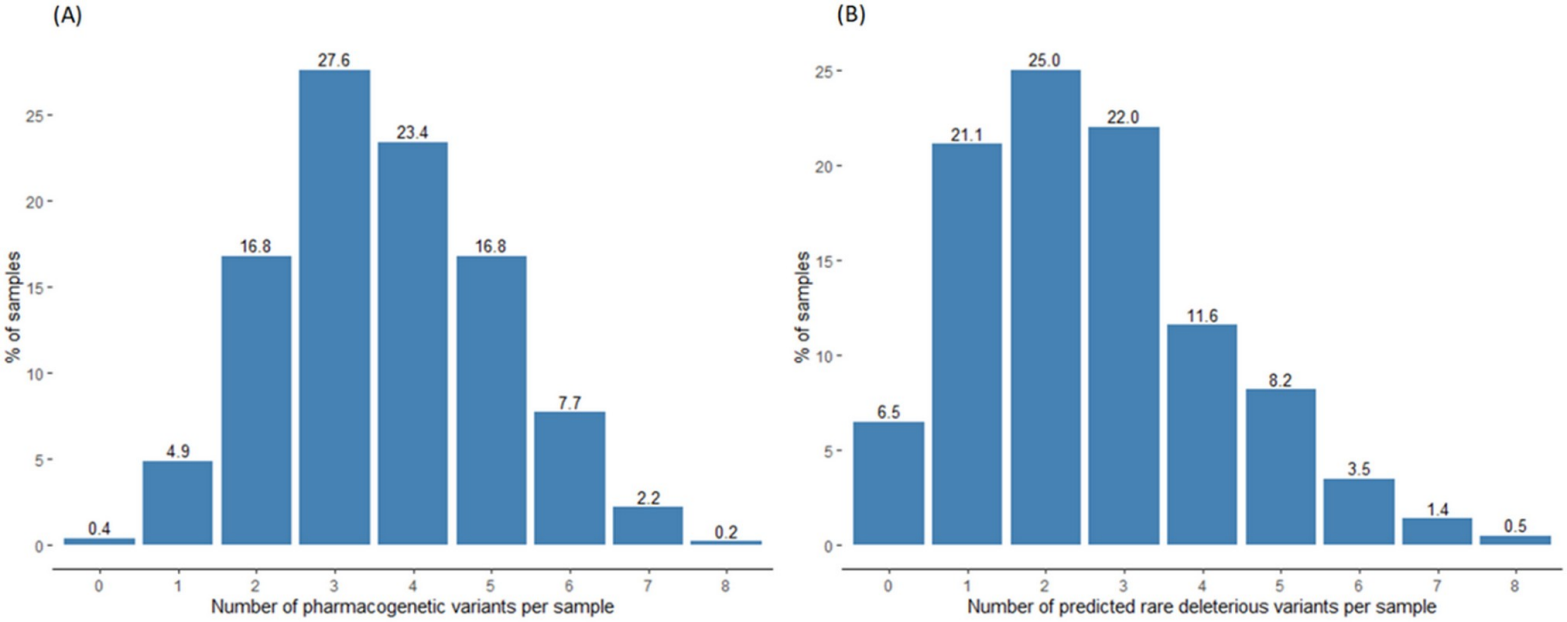
Number of pharmacogenetic variants identified in exome sequencing per sample. (A) Nearly all (99.6%) Hong Kong Chinese carried at least one known actionable pharmacogenetic variant, with a median of four variants. The highest number of actionable variants in a single sample was eight, occurring in 0.2% of samples. (B) Among 1,116 Hong Kong Chinese, 1,043 (93.5%) carried at least one rare deleterious variant in the 108 high-confidence pharmacogenes, with a median of two variants per individual. The highest number of variants observed was eight, occurring in 0.5% of the population.

The majority of the 129 known actionable pharmacogenetic variants and four HLA alleles were well-covered in the exome sequencing data, except for four variants which could not be detected by exome sequencing because they are located in non-coding regions (S2 Table). For more than 90% of the samples, depths of >8X and >30X were achieved in 121 (93.8%) and 62 (48.1%) variants, respectively (S4 Fig). In our cohort, 25 known actionable variants and all four HLA alleles were identified, accounting for 15 genes and 44 implicated drugs (S5 Table). 104 actionable variants are absent in the HK Chinese population (AF = 0). The most prevalent variant in our cohort was rs1065852 in *CYP2D6* (AF = 60.95%), a marker single nucleotide polymorphism (SNP) of a markedly reduced or null allele, while the most prevalent HLA risk allele was HLA-B*15:02 (AF = 9.68%; S6 Table). Analyzing using a per-sample approach, 1,111 (99.6%) individuals harbored at least one actionable variant, with a median of four (Fig 2A). At the gene level, *CYP2C19* (57.21%), *CYP3A5* (43.38%), and *CYP2B6* (40.51%) were the genes with the highest frequency of actionable phenotypes (Table 1). In terms of individual drugs, the antiplatelet drug clopidogrel (57.21%), immunosuppressant tacrolimus (43.38%), and anticoagulant warfarin (43.13%) had the highest frequency of actionable phenotypes (S7 Table).

**Table 1:** Frequency of actionable pharmacophenotypes in Hong Kong Chinese.

Gene	Allele	Actionable phenotype	Genotype definition	Frequency (%)	
				by phenotype	by gene
*CYP2C19*	No function: *2, *3, *6	Intermediate metabolizer	One normal function allele and one no function allele	45.25	57.21
		Poor metabolizer	Two no function alleles	11.96	
*CYP3A5*	Functional allele: *1	Extensive metabolizer	Two functional alleles	6.13	43.38
		Intermediate metabolizer	One functional allele and one nonfunctional allele	37.25	
*CYP2B6*	Decreased function: *6	Intermediate metabolizer	One normal function allele and one decreased function allele	35.28	40.51
		Poor metabolizer	Two decreased function alleles	5.23	
*CYP4F2*	rs2108622	Carrier of decreased function allele	Carrier of rs2108622 T allele	39.89	39.89
*HLA-B*	*15:02, *57:01, *58:01	*15:02 positive	Heterozygous or homozygous for HLA-B*15:02	18.42	33.88
		*57:01 positive	Heterozygous or homozygous for HLA-B*57:01	0.18	
		*58:01 positive	Heterozygous or homozygous for HLA-B*58:01	17.04	
*SLCO1B1*	rs4149056	Intermediate function	TC genotype at rs4149056	23.89	25.81
		Low function	CC genotype at rs4149056	1.92	
*NUDT15*	No function: *2, *3	Intermediate metabolizer	One normal function allele and one no function allele	17.63	18.58
		Poor metabolizer	Two no function alleles	0.95	
*IFNL3*	rs12979860	Unfavorable response genotype	Carrier of rs12979860 T allele	13.57	13.57
*CYP2D6*	Duplication of functional allele: *1x2, *1x3, *2x2, *2x3	Ultrarapid metabolizer	Carrier of duplications of functional alleles	3.3	12.24
	Reduced function: *10, *10x2, *14B, *36-*10, (*36-*10)x2, *41				
	No function: *4, *5, *6, *14A, *36, *36x2	Intermediate metabolizer	One decreased function and one no function allele	8.54	
		Poor metabolizer	Two no function alleles	0.4	
*CYP2C9*	No function: *3	Intermediate metabolizer	One normal function allele and one no function allele	5.32	5.39
		Poor metabolizer	Two no function alleles	0.07	
*UGT1A1*	Decreased function: *6, *28, *37	Poor metabolizer	Two decreased function alleles	5.08	5.08
*TPMT*	No function: *3A, *3C	Intermediate metabolizer	One normal function allele and one no function allele	2.92	2.94
		Poor metabolizer	Two no function alleles	0.02	
*HLA-A*	*31:01	*31:01 positive	Heterozygous or homozygous for HLA-A*31:01	2.48	2.48
*G6PD*	WHO class II-III: KaiPing, Canton, Gaohe, Chinese-5, Hechi, Maewo, Quing Yan	Deficient (male)	One deficient (class II–III) allele	3.68	3.68
		Deficient (female)	Two deficient (class II-III) alleles	0.14	0.14
*CFTR*	rs115545701, rs78655421	Favorable response genotype	Homozygous or heterozygous for CFTR variants listed in the FDA-approved drug label	0.09	0.09

In this study, 15 pharmacogenes were found to have actionable pharmacogenetic variants. The frequency of actionable phenotypes was derived based on the Hardy-Weinberg equation, except for *CYP2D6* where the frequency was retrieved from a published Hong Kong study by Chan et al.^25^

N/A, not applicable.

### Projected prescription impact of actionable pharmacophenotypes in HK

**Fig 3:**
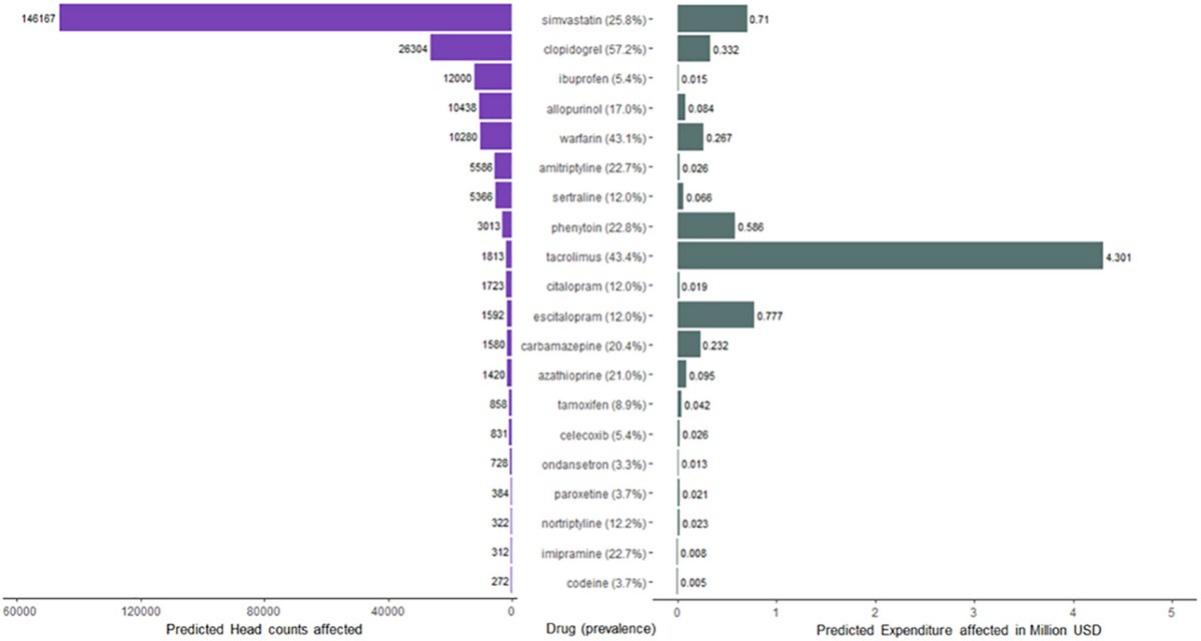
Top 20 drugs with the highest estimated prescription impact on headcount. This figure illustrates the top 20 drugs with the highest estimated prescription impact on headcount (left panel) and their respective predicted expenditure (right panel). Overall, most of the projected prescription impact was concentrated in a few drugs. The top three drugs projected to affect the greatest number of patients were the lipid-lowering drug simvastatin (146,167 patients, frequency: 25.81%), clopidogrel (26,304 patients, frequency: 57.21%), and anti-inflammatory drug ibuprofen (12,000 patients, frequency: 5.39%). It was estimated that 8,219,000 USD worth of drugs were prescribed to patients with an implicated actionable phenotype, where tacrolimus (4,301,000 USD), escitalopram (777,000 USD), and simvastatin (710,000 USD) accounted for 70.4% of the total expenditure. CPIC only recommends genotype-guided prescription of clopidogrel in patients with acute coronary syndrome receiving percutaneous coronary intervention. Indications for prescription is however not included in the CDARS system.

Based on the available prescription data in CDARS in 2019, a total of 1,006,046 HK Chinese patients had received at least one of the 36 drugs with CPIC guideline recommendations, representing 13.4% of the HK population. In general, the percentage of people prescribed with drugs with CPIC guideline recommendations increased with ages (S8 Table). For elderly patients (aged >60), 616,553 received one of the 36 drugs, accounting for 31.9% of the total elderly population. Preemptive pharmacogenetic testing is projected to have the greatest impact for simvastatin (146,167 patients, frequency: 25.81%), clopidogrel (26,304 patients, frequency: 57.21%), and ibuprofen (12,000 patients, frequency: 5.39%; Fig 3). In terms of drug costs, the 36 drugs had a total expenditure of 33,520,000 USD, which accounted for 3.6% of the annual drug expenditure of HA in the 2018–2019 fiscal year. It was estimated that 8,219,000 USD (i.e., 24.5% of drug expenditures among the 36 implicated drugs) worth of drugs were prescribed to patients with an implicated actionable phenotype. Remarkably, tacrolimus (4,301,000 USD), escitalopram (777,000 USD), and simvastatin (710,000 USD) accounted for 70.4% of the total expenditure of drugs that were prescribed to patients with an implicated actionable phenotype (Fig 3). The use of genotype-guided prescriptions was estimated to have the greatest impact for ibuprofen (1,417 patients) in the pediatric population (S5 Fig).

### Spectrum of rare, predicted deleterious variants that are potentially actionable

A total of 13,165 variants were identified in the 108 pharmacogenes and 3,586 of them (27.2%) were rare variants (gnomAD AF<1%; S4 Table). Five hundred and thirty-one rare variants were predicted to be deleterious, including 475 (89.5%) missense variants and 56 (10.5%) LoF variants (S6 Fig and S9 Table). Among the 531 rare deleterious variants, 96 (18.1%) have never been reported in public databases including dbSNP, gnomAD, and ClinVar.

Out of the 108 pharmacogenes, 97 (89.8%) had at least one deleterious variant. Genes with the highest number of rare deleterious variants included *CACNA1S* (n = 26), *CFTR* (n = 26), and *LPA* (n = 24; S7 Fig). Analyzing using a per-sample approach, 1,043 (93.5%) individuals harbored at least one rare deleterious pharmacogenetic variant, and the median was two (Fig 2B). Considering only the ten cytochrome P450 (CYP) genes, 435 (39.0%) individuals carried at least one rare deleterious variant in a CYP gene.

## Discussion

As exome sequencing becomes increasingly available, it has the potential to facilitate personalized medicine without significant additional costs. This study demonstrated the secondary use of exome data for pharmacogenetic analysis, and we found that the majority of known actionable pharmacogenetic variants and the coding regions of most pharmacogenes were well-covered. To our knowledge, this is the second study to investigate actionable pharmacogenetic variations in the Chinese population and it contained the largest sample size of Chinese subjects to date. Our data are representative of Southern Chinese, a Chinese subpopulation that is underrepresented in the literature. To the best of our knowledge, we, for the first time, have provided information on rare, predicted deleterious pharmacogenetic variants in Chinese. Additionally, the potential prescription impact of actionable pharmacogenetic variants was projected in the HK population of 7.5 million.

### Burden and projected prescription impact of known actionable pharmacogenetic variations

**Fig 4:**
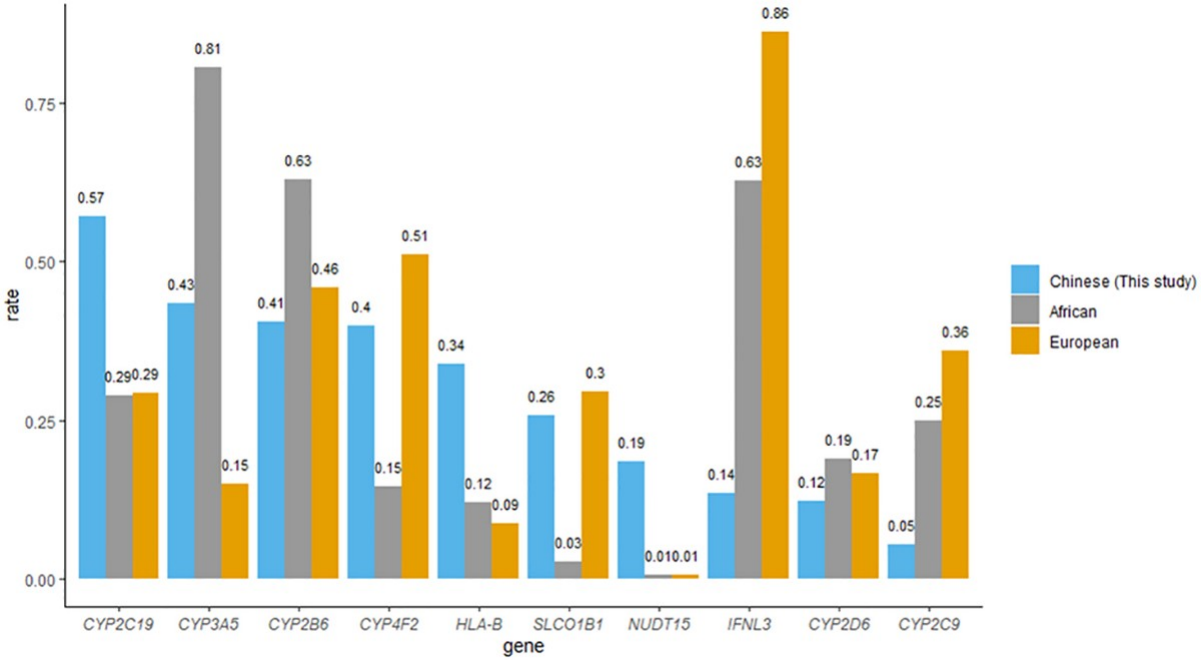
Frequency of the top ten actionable phenotypes in Hong Kong Chinese compared to that of Africans and Europeans. This figure compares the top ten actionable phenotypes in Hong Kong Chinese with that of Africans and Europeans. The actionable phenotype frequency of Africans and Europeans was retrieved from data from Chanfreau-Coffinier et al., Walker et al. and supplementary information in the CPIC guideline [30,36]. Actionable phenotypes include *CYP2C19* IM and PM; *CYP3A5* EM and IM; *CYP2B6* IM and PM; carrier of *CYP4F2* decreased function allele; carrier of HLA-B*15:02; *57:01 and *58:01; *SLCO1B1* intermediate and low-function diplotypes; *NUDT15* IM and PM; *IFNL3* unfavorable response genotype; *CYP2D6* UM, IM, and PM; and *CYP2C9* IM and PM. CYP2C19*17 is not readily detected in exome sequencing and therefore *CYP2C19* RM and UM were not included. The frequency of actionable phenotypes in *CYP2C19*, *HLA-B*, and *NUDT15* was found to be higher in Hong Kong Chinese than in Europeans and Africans. In contrast, actionable genotypes in *CYP3A5*, *CYP2B6*, and *CYP2D6* were more prevalent in Africans, whereas actionable phenotypes in *CYP4F2*, *SLCO1B1*, *IFNL3*, and *CYP2C9* were more prevalent in Europeans; however, all of these genes were within the top ten actionable phenotypes in Hong Kong. IM, intermediate metabolizer; PM, poor metabolizer; EM, extensive metabolizer; UM, ultrarapid metabolizer; RM, rapid metabolizer.

Consistent with other populations, nearly all HK Chinese (99.6%) harbored at least one pharmacogenetic variant, with a median of four variants [7,8]^.^ Nonetheless, the spectrum of actionable genotypes was different compared to that of African and European populations (Fig 4). The highest actionable phenotype in Europeans and Africans was *IFNL3* and *CYP3A5*, respectively, which had a frequency of more than 80% in their respective populations; however, less than half of the HK Chinese carried an actionable phenotype in these genes. In contrast, while *NUDT15* ranked seventh among the drugs with highest actionable phenotypes in HK Chinese (frequency: 18.58%), only 1% of Europeans and Africans carried actionable phenotypes in this gene. It is therefore more important to consider defective *NUDT15* alleles when azathioprine is prescribed in the Chinese population. In contrast to *NUDT15*, which is the key determinant of azathioprine-induced myelosuppression in Asians, defective *TPMT* alleles should be considered in Europeans instead [28,29]. This suggests that even when the same drug is prescribed, different pharmacogenes should be considered in each population. Furthermore, considering the same pharmacogene, the alleles contributing to actionable phenotypes could be different across different populations. For example, while *G6PD* deficiency is common in Africans and Chinese, the A allele alone explains nearly all *G6PD* deficiencies in Africans, whereas seven *G6PD* alleles contribute to the deficiency in HK Chinese [30].

Based on prescription data of the HK public healthcare system, 13.4% of the HK population (1,006,046 patients) received at least one of the 36 drugs with CPIC guideline recommendations. The total expenditure on CPIC actionable drugs in 2019 was 33,520,000 USD, and it was estimated that 8,219,000 USD (24.5%) worth of drugs were prescribed to subjects with an implicated actionable phenotype. Pharmacogenetic results would improve patient care and allocation of resources. For example, in order to reduce the risk of thiopurine-related myelosuppression, 1,354 (17.63%) patients taking azathioprine would require a starting dose reduction of 30–80%, and 66 (0.95%) patients would require alternative medications. Another example is the lipid-lowering drug simvastatin, which was found to have the greatest prescription impact in terms of headcount in our study. A previous study on *SLCO1B1* reported an 18% cumulative risk of simvastatin-induced myopathy for the CC genotype (frequency in HK: 1.92%) and a 3% cumulative risk for the CT genotype (frequency in HK: 23.89%) [31]. Projecting from the frequency of actionable phenotype derived from our dataset onto the CDARS prescription data, it was estimated that 146,167 patients prescribed with simvastatin had an actionable phenotype in 2019. If all of these patients prescribed with a lower dose of simvastatin or another statin, it was estimated that 6,019 cases of simvastatin-induced myopathy can be prevented. Dosing can also be increased based on genotype-guided prescription. Tacrolimus, an immunosuppressive drug used by organ transplant recipients to lower the risk of organ rejection, was predicted to affect 1,813 (43.38%) people, accounting for the highest expenditure (4,301,000 USD) among all the CPIC actionable drugs in HK. Based on the pharmacogenetic results, these 1,813 people required a 1.5 to 2 times increase in starting dose, further increasing the prescription cost. Nevertheless, genotype-guided prescription in tacrolimus is known to achieve therapeutic concentrations earlier and had fewer out-of-range concentrations when compared to standard dosing [32]. With expansion of the CPIC guidelines to include other commonly prescribed drugs such as tramadol and proton pump inhibitors, the prescription impact of pharmacogenetic testing is likely to increase in the near future.

### Spectrum of rare, deleterious pharmacogenetic variants

The effect of rare pharmacogenetic variants on drug responses should not be neglected, since they may account for nearly all inter-individual variabilities in more than half of the pharmacogenes [11]. At present, pharmacogenetic testing is performed by SNP arrays targeting specific alleles and hence, the detection of rare variants is impossible. In contrast, exome or genome sequencing would result in an abundance of rare variants, but determining the effect of rare variants on drug responses is difficult without functional data [19]. In the present study, we aimed to maximize the discovery of potentially deleterious pharmacogenetic variants. We found that 93.5% of subjects carried at least one rare, deleterious pharmacogenetic variant, with a median of two variants. This suggests that despite being individually rare, pharmacogenetic variants with AF <1% are collectively common. The rare, deleterious pharmacogenetic variants reported in this study can be prioritized for functional studies, especially for variants with consensus deleterious effects predicted across multiple bioinformatics tools and with known gene mechanisms. An example would be the *CYP2C9* splice variant c.1291+1G>T, which was predicted to be deleterious by both CADD and LOFTEE. Although this variant has not been reported in PharmVar, other LoF variants in *CYP2C9* have been reported as “no function” [26]. In the future, saturation mutagenesis studies will likely aid in determining how deleterious rare pharmacogenetic variants are [33].

### Study limitations

First, due to the technical limitations of exome sequencing, non-coding regions, copy number variations, structural variations, and loci with high genomic complexity either were poorly/not covered with exome sequencing or were difficult to detect with bioinformatics. For example, four actionable variants located in non-coding regions were not sequenced, and four variants, UGT1A1*28, *IFNL3* rs12979860, *CYP2C19* rs4244285 and *CYP3A5* rs776746, were sequenced in less than 70% of the samples (S2 Table). The reported AF of these variants should therefore be interpreted with caution. Advancements in bioinformatics may have overcome some technical difficulties, such as using HLA-HD in this study for HLA typing. The AF of HLA identified in this study agreed well with that of the HK Bone Marrow Donor Registry (S6 Table), suggesting the accuracy of HLA-HD [34]. In the future, copy number variations and structural variations could be more reliably identified from exome data with bioinformatics. The second limitation is related to the projected impact of preemptive pharmacogenetic testing. The prescription data of private medical practitioners and over-the-counter medications were not available in this study. Therefore, our analysis was limited to prescription data in the public healthcare setting. Although our data were limited to public hospitals, the public healthcare system accounted for approximately 90% of all secondary and tertiary health services in HK. Lastly, indications for drug prescription could not be retrieved from the CDARS database. The CPIC guideline only recommended *CYP2C19* genotype-guided prescription of clopidogrel in patients with acute coronary syndrome receiving anti-platelet therapy for percutaneous coronary intervention, therefore the projected impact of clopidogrel could be over-estimated [35].

In conclusion, nearly all individuals carried at least one actionable pharmacogenetic variant and one rare, deleterious pharmacogenetic variant in our cohort. It was estimated that one-seventh of the HK population received at least one of the 36 drugs with CPIC guideline recommendations, and 8,219,000 USD worth of drugs were prescribed to patients with an implicated actionable phenotype. This indicates that preemptive pharmacogenetic testing has the potential to improve patient care and allocation of resources significantly.

## Supporting information

S1 Text Supplementary Methods. (DOCX)

S1 Table Sequencing platform and capture kit used in the Hong Kong Chinese exome sequencing data. (XLSX)

S2 Table List of the 133 actionable pharmacogenetic variants/alleles. ^#^ Call rate refers to the % of samples having coverage ≥8X in the exome sequencing cohort. ADR, adverse drug reaction; PK, pharmacokinetics.
(XLSX)

S3 Table List of the 108 high confidence pharmacogenes. (XLSX)

S4 Table Spectrum of variants identified in the 108 high-confidence pharmacogenes. (XLSX)

S5 Table Actionable pharmacogenetic variants identified in the Hong Kong Chinese exome sequencing cohort. ^#^ Call rate refers to the % of samples having coverage ≥8X in the exome sequencing cohort. ^^^ *4, *10, *36, *47, *49, *54, *56, *57, *69, *72, *99, *100, *101, *114. AF_HK_, allele frequency in Hong Kong; LoF, loss-of-function; NSAID, non-steroidal anti-inflammatory drug (includes celecoxib, ibuprofen, and piroxicam); SSRI, selective serotonin reuptake inhibitor (includes citalopram, escitalopram, and sertraline);TCA, tricyclic antidepressant (includes amitriptyline, clomipramine, doxepin, imipramine, and trimipramine).
(XLSX)

S6 Table Table of actionable HLA risk alleles identified in the Hong Kong Chinese exome sequencing cohort. AF_HK_, allele frequency in Hong Kong. ^a^Data from Hong Kong Bone Marrow Donor Registry (HKBMDR)[34]; ^b^Data from Chinese Bone Marrow Donor Program (CBMD)[37].
(XLSX)

S7 Table Frequency of actionable phenotype(s) and projected impact for each drug. ^a^Carrier rate of *G6PD* deficiency projected from male-female ratio of 45.6:54.4 in Hong Kong. ^b^Variants include p.(Arg74Trp) and p.(Arg117His). Cystic fibrosis is uncommon in Chinese, hence Ivacaftor is not prescribed in public healthcare systems. UM, ultrarapid metabolizer; IM, intermediate metabolizer; EM, extensive metabolizer; PM, poor metabolizer.
(XLSX)

S8 Table The number and percentage of people prescribed with one of the 36 drugs with CPIC guideline recommendation in different age group. Based on the available prescription data in CDARS in 2019, a total of 1,006,046 Hong Kong Chinese patients had received at least one of the 36 drugs with CPIC guideline recommendations, representing 13.4% of the Hong Kong population. The percentage of people prescribed with one of the 36 drugs with CPIC guideline recommendations increased with age.
(XLSX)

S9 Table List of 531 rare, deleterious variants identified in this study. (XLSX)

S1 Fig Analysis flowchart of this study. Concatenated exome sequencing data was first run through sample-, variant-, and genotype-level quality control (QC) procedures. In the analysis of known actionable pharmacogenetic variants, we first extracted data based on a curated list of 129 variants and four HLA alleles, and subsequently projected the prescription impact in the Hong Kong public healthcare system. We further processed the dataset for analysis of rare variants of the 108 high-confidence pharmacogenes. The final list of rare, predicted deleterious variants included only missense and loss-of-function (LoF) variants with gnomAD allele frequency (AF) <1% and at least one deleterious prediction by bioinformatics algorithm.
(TIF)

S2 Fig Coverage of the coding regions of the 108 high-confidence pharmacogenes. In general, exome sequencing covered the 108 high-confidence pharmacogenes well, with 104 of them having a mean coverage of at least 8X in over 75% of the samples. Genes that did not have a mean coverage of 8X included *CCHCR1*, *TNF*, *IFNL4*, and *GSTM1*. Mean coverages of 30X and 50X were achieved in over 75% of samples for 90 and 31genes, respectively.
(TIF)

S3 Fig Spectrum and functional consequence of variants identified in the 108 high-confidence pharmacogenes. A total of 13,165 variants were identified in the108 high-confidence pharmacogenes, with 10,192 (77.4%) being intronic variants. Coding variants accounted for 1,719 of the variants, with the majority (58.6%) being nonsynonymous variants and 2.5% being loss-of-function (frameshift, stop-gain, and start-loss) variants. SNV, single-nucleotide variant; UTR3, 3′ untranslated region; UTR5, 5′ untranslated region.
(TIF)

S4 Fig Cumulative known actionable variant count by proportion of samples with specified coverage. In our cohort, >8X read depth and >30X read depth were achieved in >90% of samples in 121/129 (93.8%) and 62/129 (48.1%) known actionable variants, respectively.
(TIF)

S5 Fig Top 20 drugs with the highest estimated prescription impact on headcount in the pediatric population (age < 19). The top three drugs with the highest pharmacogenetic impact in the pediatric population (age <19) based on headcount were ibuprofen (1417 patients, frequency:5.39%), atomoxetine (235 patients, frequency:12.24%), and sertraline (156 patients, frequency:11.96%). The top three drugs with highest pharmacogenetic impact based on expenditures were tacrolimus (238,000 USD), atomoxetine (48,000 USD), and escitalopram (32,000 USD).
(TIF)

S6 Fig Venn diagrams showing the overlapping deleterious predictions using different bioinformatics tools. (A) Among the 829 rare (gnomAD global AF <1%) missense variants in the 108 high-confidence pharmacogenes, 475 variants were predicted to be deleterious by at least one of the three bioinformatics tools (CADD, REVEL, and PREDICT), and 89 variants had consensus deleterious predictions. There were 354 rare missense variants that were not predicted as deleterious by all of the bioinformatics tools. (B) Among the 63 rare LoF variants, 56 were predicted to be deleterious by either CADD or LOFTEE, and 43 variants had consensus deleterious predictions. There were 7 rare LoF variants that were not predicted as deleterious by both of the bioinformatics tools. AF, allele frequency; LoF, loss-of-function.
(TIF)

S7 Fig Manhattan plot of gene distribution among rare, predicted deleterious variants. The Manhattan plot summarizes the gene distribution of 531 rare, predicted deleterious variants identified and their chromosome number (chr) in the human genome. *CACNA1S* (n = 26), *CFTR* (n = 26), and *LPA* (n = 24) had the highest number of rare deleterious variants among the 108 high-confidence pharmacogenes. LoF, loss-of-function.
(TIF)
