# Supplementary material for: Actionable pharmacogenetic variants in Hong Kong Chinese exome sequencing data and projected prescription impact in the Hong Kong population
Source: PLoS Genet. 2021 Feb 18;17(2):e1009323. doi: 10.1371/journal.pgen.1009323 (PMC7891783; doi:10.1371/journal.pgen.1009323)
Supplement: S1 Text — (DOCX) [file pgen.1009323.s001.docx]

**Supplementary Methods**

**Date of retrieval or release version of accessed data/ software**

| **Accessed data/ software** | **Version or date of access** |
| --- | --- |
| PharmGKB | January 23, 2020 |
| CPIC | April 1, 2020 |
| PharmVar | December 27, 2019 |
| dbSNP | Build 140 |
| 1000 Genomes Project | Phase 3 data |
| gnomAD (gnomad.broadinstitute.org) | Version 2.1.1 |
| Chinese Genomic Variation Database (https://bigd.big.ac.cn/cgvd/) | Version 1.0 |
| ClinVar | Version 20190305 |
| Ensembl | Release 99 |
| Reference genome | UCSC hg19 reference genome assembly |
| Genome analysis toolkit (GATK) | Version 3.4 |
| Burrows-Wheeler Aligner | Version 0.7.8 |
| Picard | Version 1.79 |
| HLA-HD | Version 1.2.0 |
| Variant Tools (vtools) | Version 2.7.0 |
| wANNOVAR | Build 20200223 |
| R | Version 3.6.0 |

**Subject recruitment, exome sequencing and bioinformatics analysis**

The raw exome sequencing data of 1141 samples were processed by pipeline based on GATK.^[1]^ Reads were aligned to the University of California Santa Cruz (UCSC) hg19 reference genome assembly by Burrows-Wheeler Aligner and duplicated reads were removed by Picard.^[2]^ Local realignment around indels, base quality score recalibration, and cohort-based multi-sample variant calling were performed using the GATK toolset. HLA typing for the targeted HLA genes (*HLA-A* and *HLA-B*) was performed using HLA-HD.^[3]^

Stringent quality control (QC) procedures were performed on the exome sequencing dataset. Multiple QC steps at sample levels were performed. First, all samples were verified to have a FREEMIX score of < 0.05 (mean = 0.001794, median = 0.0008) using VerifyBamID to detect sample contamination.^[4]^ Second, a sample check was performed using Peddy and 17 duplicated or related samples were identified and subsequently removed from further analysis. Third, principal component analysis was performed using Peddy to compare with 1000 Genomes Project reference data.^[4],[5]^ Samples not clustering with the East Asian population (n = 8) were removed from analysis (Fig 1). At variant level QC, variants failing any of the following filters were removed from downstream analysis by KGGSeq: genotyping quality <20, read depth <8X, and Hardy-Weinberg test P-value ≤ 1×10^-5^.^[6]^ Finally, all variants also needed to pass Variant Quality Score Recalibration (VQSR) annotated by GATK with the SNP tranche sensitivity threshold of 99.5% and INDEL tranche sensitivity threshold of 99.0%.

Variants passing the QC were loaded into our in-house genome database powered by Variant Tools (vtools).^[7]^ Variants of the108 high-confidence pharmacogenes were extracted from vtools and annotated by wANNOVAR,^[8]^ including data from dbSNP, the gnomAD dataset, the Clinvar database, and the Chinese Genomic Variation Database.^[9],[10]^

Variants were categorized into four classes according to their allele frequency (AF): common (>5%), low frequency (1% to 5%), rare (0.1% to 1%), and very rare (<0.1%) based on the global AF of the gnomAD database. In this study, a rare variant was defined as a variant having a gnomAD global AF <1%. Currently, the pharmacogenetics community has not reached a consensus on standardized methodology to evaluate the functional impact of rare variants. In this study, CADD, REVEL, and PREDICT were used to predict the consequence of missense variants identified in the 108 high-confidence pharmacogenes.^[11],[12],[13]^ A missense variant was considered deleterious when it had a Phred-scaled CADD score >20, REVEL score >0.7, or PREDICT score >0.6. For loss-of-function (LoF) variants, CADD and LOFTEE were used to examine the deleterious effect, and an LoF variant was considered deleterious when it had a Phred-scaled CADD score >20 or LOFTEE of “high-confidence.” The gene lengths of the 108 pharmacogenes were retrieved from Ensembl based on the UCSC hg19 reference genome assembly.^[14]^

**Projected prescription impact analysis**

In the case of a drug affected by more than one pharmacogene, the actionable phenotype frequency is calculated as follows to avoid double-counting individuals who have an actionable phenotype in more than one gene.

$$Total actionable phenotype frequency=F_{A}+F_{B}-F_{A}\times F_{B}$$

Where $F_{A}$and $F_{B}$ refers to the actionable phenotype frequency of gene A and gene B respectively.

**Figures**

Fig 1 - Principal component analysis to evaluate the ethnicity of the exome sequencing data


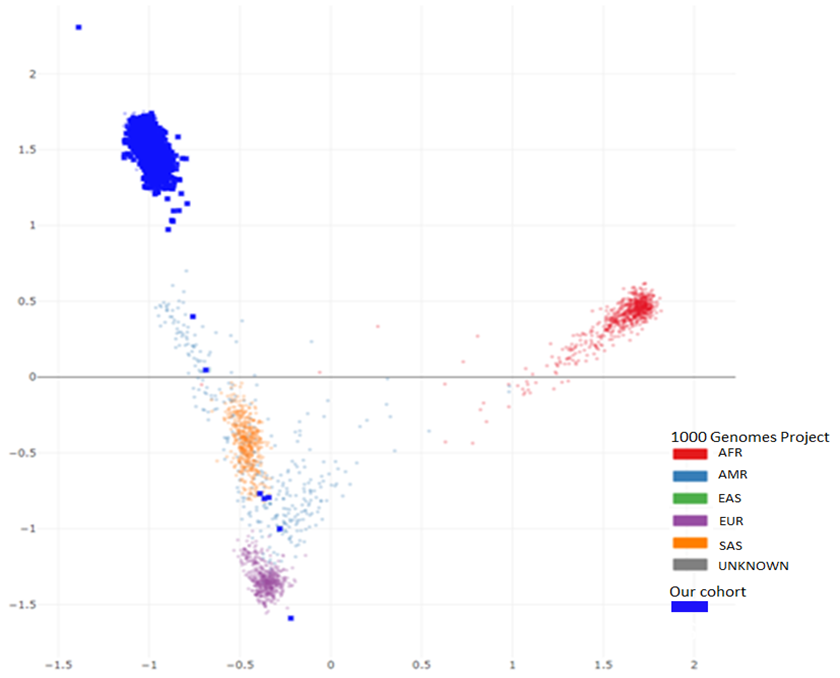


Principal component analysis shows that 1116 of our samples clustered with the East Asian samples from the 1000 Genome project (in the top left corner), and eight samples were removed from the final dataset as they clustered with other populations.
AFR, African; AMR, Ad Mixed American; EAS, East Asian; EUR, European; SAS, South Asian

**Supplementary References**

1. Poplin R, Ruano-Rubio V, DePristo MA, Fennell TJ, Carneiro MO, Van der Auwera GA, et al. Scaling accurate genetic variant discovery to tens of thousands of samples. bioRxiv. 2018:201178. doi: 10.1101/201178.

2. Li H, Durbin R. Fast and accurate short read alignment with Burrows-Wheeler transform. Bioinformatics. 2009;25(14):1754-60. Epub 2009/05/20. doi: 10.1093/bioinformatics/btp324. PubMed PMID: 19451168; PubMed Central PMCID: PMCPMC2705234.

3. Kawaguchi S, Higasa K, Shimizu M, Yamada R, Matsuda F. HLA-HD: An accurate HLA typing algorithm for next-generation sequencing data. Hum Mutat. 2017;38(7):788-97. Epub 2017/04/19. doi: 10.1002/humu.23230. PubMed PMID: 28419628.

4. Jun G, Flickinger M, Hetrick KN, Romm JM, Doheny KF, Abecasis GR, et al. Detecting and estimating contamination of human DNA samples in sequencing and array-based genotype data. Am J Hum Genet. 2012;91(5):839-48. Epub 2012/10/30. doi: 10.1016/j.ajhg.2012.09.004. PubMed PMID: 23103226; PubMed Central PMCID: PMCPMC3487130.

5. Auton A, Brooks LD, Durbin RM, Garrison EP, Kang HM, Korbel JO, et al. A global reference for human genetic variation. Nature. 2015;526(7571):68-74. Epub 2015/10/04. doi: 10.1038/nature15393. PubMed PMID: 26432245; PubMed Central PMCID: PMCPMC4750478.

6. Li MX, Kwan JS, Bao SY, Yang W, Ho SL, Song YQ, et al. Predicting mendelian disease-causing non-synonymous single nucleotide variants in exome sequencing studies. PLoS Genet. 2013;9(1):e1003143. Epub 2013/01/24. doi: 10.1371/journal.pgen.1003143. PubMed PMID: 23341771; PubMed Central PMCID: PMCPMC3547823.

7. San Lucas FA, Wang G, Scheet P, Peng B. Integrated annotation and analysis of genetic variants from next-generation sequencing studies with variant tools. Bioinformatics. 2012;28(3):421-2. Epub 2011/12/06. doi: 10.1093/bioinformatics/btr667. PubMed PMID: 22138362; PubMed Central PMCID: PMCPMC3268240.

8. Yang H, Wang K. Genomic variant annotation and prioritization with ANNOVAR and wANNOVAR. Nat Protoc. 2015;10(10):1556-66. Epub 2015/09/18. doi: 10.1038/nprot.2015.105. PubMed PMID: 26379229; PubMed Central PMCID: PMCPMC4718734.

9. Karczewski KJ, Francioli LC, Tiao G, Cummings BB, Alföldi J, Wang Q, et al. Variation across 141,456 human exomes and genomes reveals the spectrum of loss-of-function intolerance across human protein-coding genes. bioRxiv. 2019:531210. doi: 10.1101/531210.

10. Du Z, Ma L, Qu H, Chen W, Zhang B, Lu X, et al. Whole Genome Analyses of Chinese Population and De Novo Assembly of A Northern Han Genome. Genomics Proteomics Bioinformatics. 2019;17(3):229-47. Epub 2019/09/09. doi: 10.1016/j.gpb.2019.07.002. PubMed PMID: 31494266; PubMed Central PMCID: PMCPMC6818495.

11. Rentzsch P, Witten D, Cooper GM, Shendure J, Kircher M. CADD: predicting the deleteriousness of variants throughout the human genome. Nucleic Acids Res. 2019;47(D1):D886-d94. Epub 2018/10/30. doi: 10.1093/nar/gky1016. PubMed PMID: 30371827; PubMed Central PMCID: PMCPMC6323892.

12. Ioannidis NM, Rothstein JH, Pejaver V, Middha S, McDonnell SK, Baheti S, et al. REVEL: An Ensemble Method for Predicting the Pathogenicity of Rare Missense Variants. Am J Hum Genet. 2016;99(4):877-85. Epub 2016/09/27. doi: 10.1016/j.ajhg.2016.08.016. PubMed PMID: 27666373; PubMed Central PMCID: PMCPMC5065685.

13. Zhou Y, Mkrtchian S, Kumondai M, Hiratsuka M, Lauschke VM. An optimized prediction framework to assess the functional impact of pharmacogenetic variants. Pharmacogenomics J. 2019;19(2):115-26. Epub 2018/09/13. doi: 10.1038/s41397-018-0044-2. PubMed PMID: 30206299; PubMed Central PMCID: PMCPMC6462826 that they have no conflict of interest.

14. Hunt SE, McLaren W, Gil L, Thormann A, Schuilenburg H, Sheppard D, et al. Ensembl variation resources. Database (Oxford). 2018;2018. Epub 2018/12/24. doi: 10.1093/database/bay119. PubMed PMID: 30576484; PubMed Central PMCID: PMCPMC6310513.
