# Supplementary figures and images for: Actionable pharmacogenetic variants in Hong Kong Chinese exome sequencing data and projected prescription impact in the Hong Kong population
Source: PLoS Genet. 2021 Feb 18;17(2):e1009323. doi: 10.1371/journal.pgen.1009323 (PMC7891783; doi:10.1371/journal.pgen.1009323)

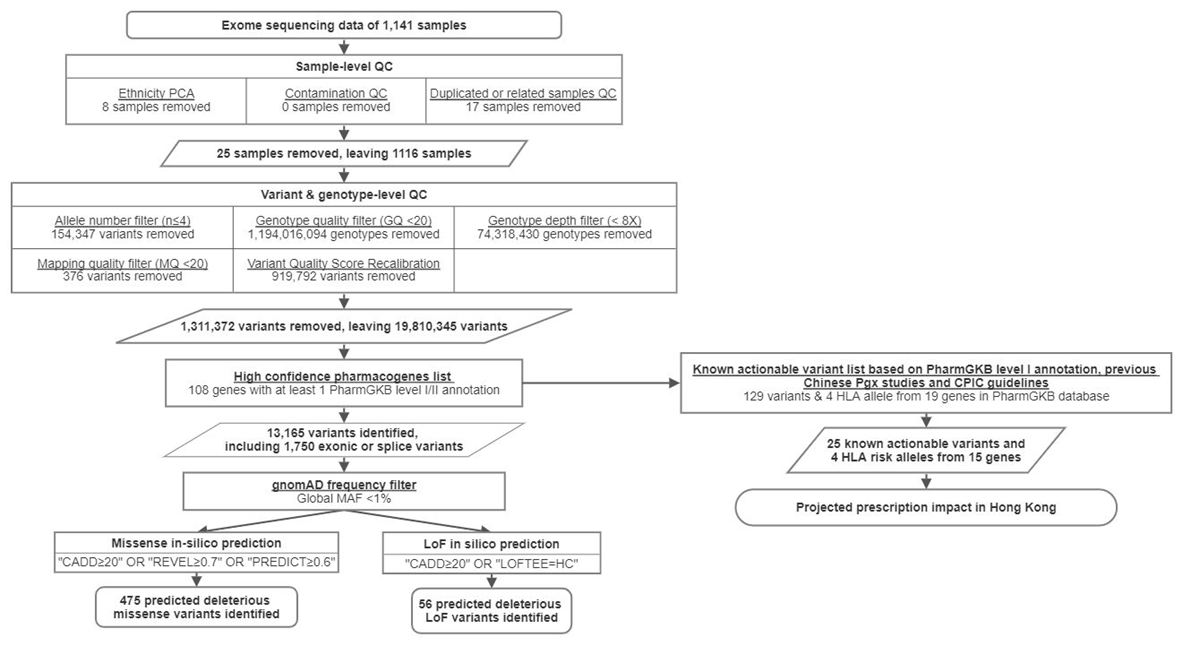

Supplement: S1 Fig — Concatenated exome sequencing data was first run through sample-, variant-, and genotype-level quality control (QC) procedures. In the analysis of known actionable pharmacogenetic variants, we first extracted data based on a curated list of 129 variants and four HLA alleles, and subsequently projected the prescription impact in the Hong Kong public healthcare system. We further processed the dataset for analysis of rare variants of the 108 high-confidence pharmacogenes. The final list of rare, predicted deleterious variants included only missense and loss-of-function (LoF) variants with gnomAD allele frequency (AF) <1% and at least one deleterious prediction by bioinformatics algorithm. (TIF) [file pgen.1009323.s011.tif]

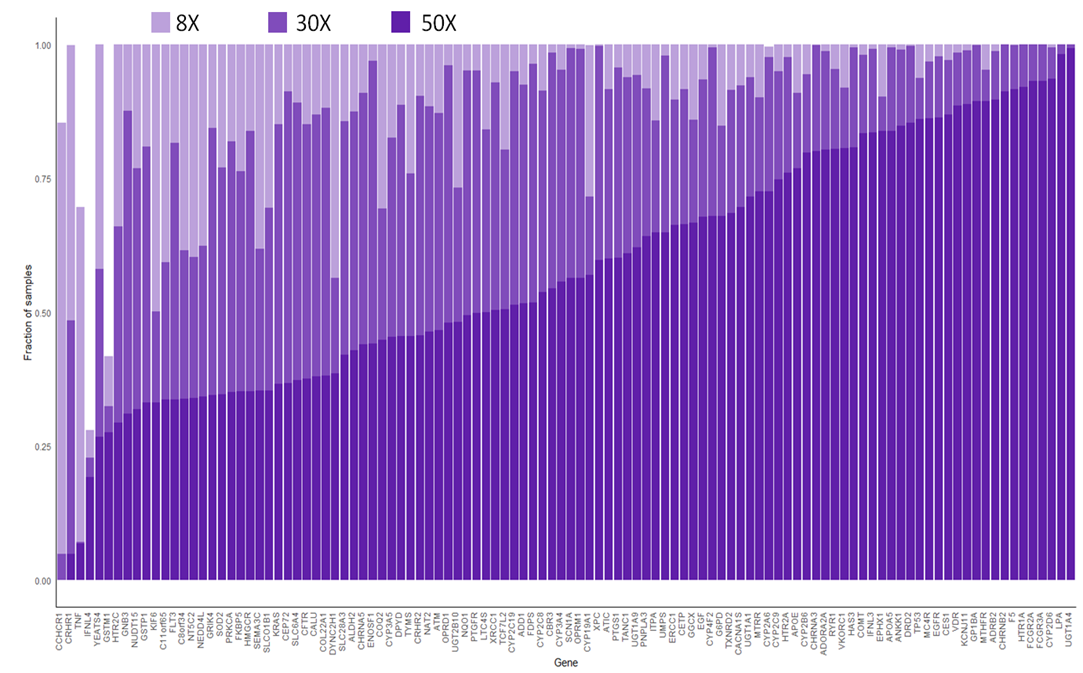

Supplement: S2 Fig — In general, exome sequencing covered the 108 high-confidence pharmacogenes well, with 104 of them having a mean coverage of at least 8X in over 75% of the samples. Genes that did not have a mean coverage of 8X included CCHCR1, TNF, IFNL4, and GSTM1. Mean coverages of 30X and 50X were achieved in over 75% of samples for 90 and 31genes, respectively. (TIF) [file pgen.1009323.s012.tif]

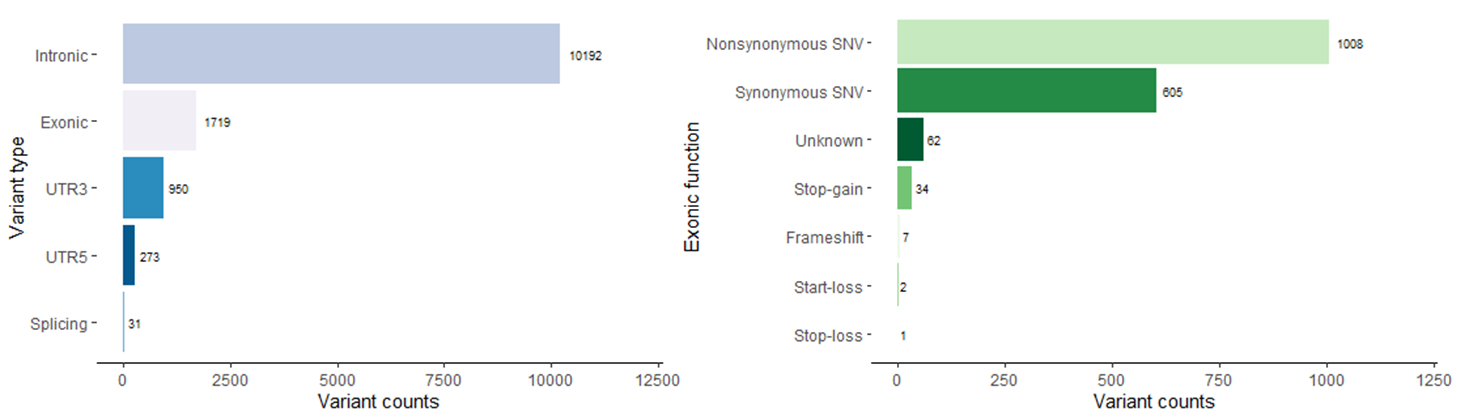

Supplement: S3 Fig — A total of 13,165 variants were identified in the108 high-confidence pharmacogenes, with 10,192 (77.4%) being intronic variants. Coding variants accounted for 1,719 of the variants, with the majority (58.6%) being nonsynonymous variants and 2.5% being loss-of-function (frameshift, stop-gain, and start-loss) variants. SNV, single-nucleotide variant; UTR3, 3′ untranslated region; UTR5, 5′ untranslated region. (TIF) [file pgen.1009323.s013.tif]

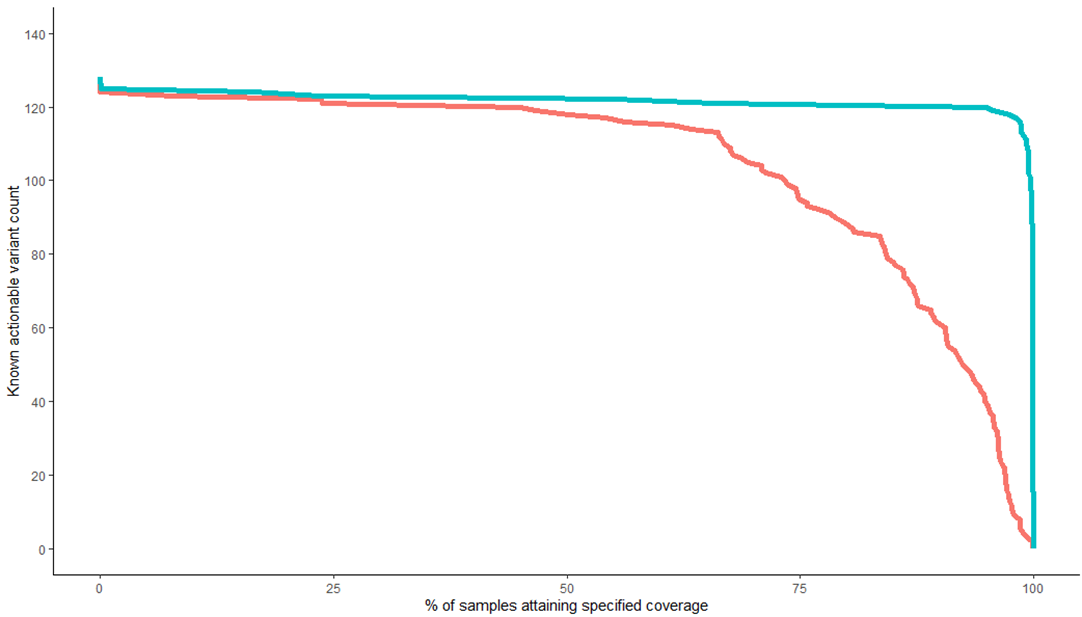

Supplement: S4 Fig — In our cohort, >8X read depth and >30X read depth were achieved in >90% of samples in 121/129 (93.8%) and 62/129 (48.1%) known actionable variants, respectively. (TIF) [file pgen.1009323.s014.tif]

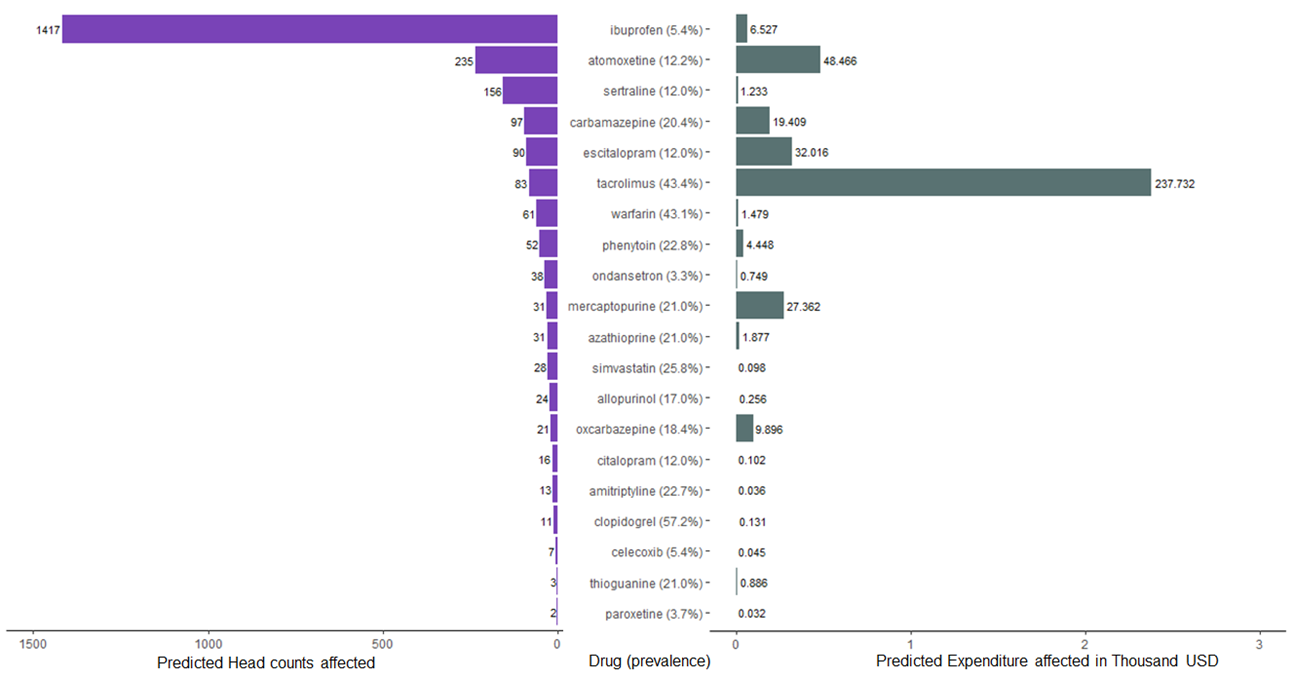

Supplement: S5 Fig — The top three drugs with the highest pharmacogenetic impact in the pediatric population (age <19) based on headcount were ibuprofen (1417 patients, frequency:5.39%), atomoxetine (235 patients, frequency:12.24%), and sertraline (156 patients, frequency:11.96%). The top three drugs with highest pharmacogenetic impact based on expenditures were tacrolimus (238,000 USD), atomoxetine (48,000 USD), and escitalopram (32,000 USD). (TIF) [file pgen.1009323.s015.tif]

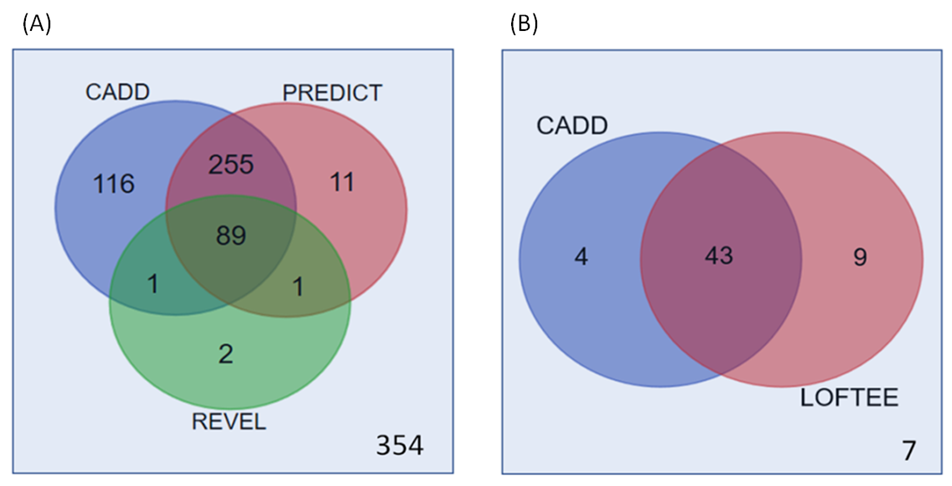

Supplement: S6 Fig — (A) Among the 829 rare (gnomAD global AF <1%) missense variants in the 108 high-confidence pharmacogenes, 475 variants were predicted to be deleterious by at least one of the three bioinformatics tools (CADD, REVEL, and PREDICT), and 89 variants had consensus deleterious predictions. There were 354 rare missense variants that were not predicted as deleterious by all of the bioinformatics tools. (B) Among the 63 rare LoF variants, 56 were predicted to be deleterious by either CADD or LOFTEE, and 43 variants had consensus deleterious predictions. There were 7 rare LoF variants that were not predicted as deleterious by both of the bioinformatics tools. AF, allele frequency; LoF, loss-of-function. (TIF) [file pgen.1009323.s016.tif]

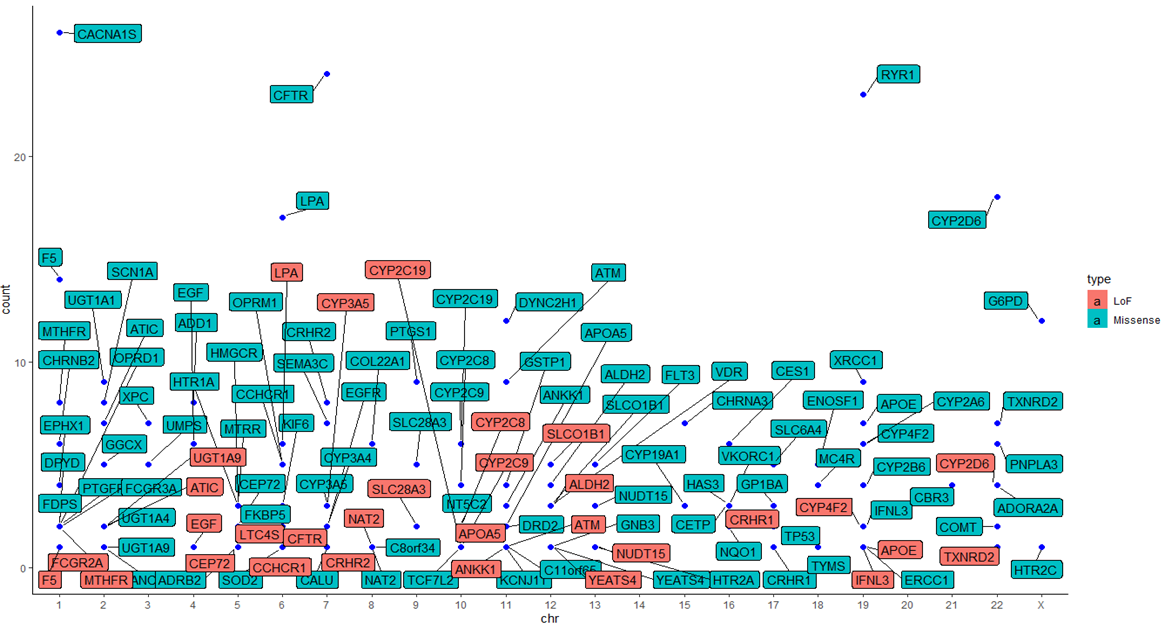

Supplement: S7 Fig — The Manhattan plot summarizes the gene distribution of 531 rare, predicted deleterious variants identified and their chromosome number (chr) in the human genome. CACNA1S (n = 26), CFTR (n = 26), and LPA (n = 24) had the highest number of rare deleterious variants among the 108 high-confidence pharmacogenes. LoF, loss-of-function. (TIF) [file pgen.1009323.s017.tif]
